# Clinical and paraclinical aspects of infectious mononucleosis

**DOI:** 10.1186/1471-2334-13-S1-P65

**Published:** 2013-12-16

**Authors:** Sonia Drăghici, Andrei Csep

**Affiliations:** 1 Faculty of Medicine and Pharmacy, University of Oradea, Romania

## Background

Infectious mononucleosis has polymorphous clinical aspects. In children, primary Epstein-Barr virus (EBV) infection is often mild or asymptomatic. In adults, the enlargement of lymph nodes, fever and angina may be associated with diverse complications.

## Methods

We studied the clinical and paraclinical features of 176 patients with infectious mononucleosis admitted in our hospital in the last 2 years (2011-2012). We emphasized the clinical aspects of the cases.

## Results

The incidence of infectious mononucleosis was highest in the age group 15 to 24. The age of the patient has a profound influence on the clinical expression of EBV infection. The incidence of EBV infection is higher in urban environment, where the interhuman contact is closer, and also the addressability to health-care services is better and the laboratory diagnosis is more easily available to the population. The clinical forms of the disease have been grouped according to the severity of illness into the following: light forms – 44 cases (25%), medium forms – 122 cases (69%), and severe forms – 10 cases (6%). A number of 64 cases, considered severe forms, presented mononucleosic hepatitis (36%), 112 cases (64%) were anicteric. 75 cases (42.6%) presented disorders of protein synthesis, factors of coagulation and albumin, hypoglycemia, and dyslipidemia.

The most frequent complications during the acute EBV infection were: hepatic (64 cases – 36%); hematologic (19 cases – 11%) - anemia, thrombocytopenia; neurologic (5 cases – 3%) - meningitis, neuritis; pulmonary (2 cases – 1%) - interstitial pulmonary infiltrate, and chronic fatigue syndrome (7 cases – 4%). The therapy consisted in drugs for sustaining the liver functions, corticotherapy accompanied by antibiotic-therapy in the 10 severe cases of central nervous system involvement (15%) and in 15 other cases with hematologic or other complications (20%). Altogether, the combination was utilized in 25 cases (47%), while the rest of 151 cases (65%) didn’t receive corticotherapy.

## Conclusions

The evolution and prognosis of acute EBV infection is closely connected with the peculiar clinical forms and complications of the disease.

